# UV-LED Curable Acrylic Films Containing Phosphate Glass Powder: Effect of the Filler Loading on the Thermal, Optical, Mechanical and Flame Retardant Properties

**DOI:** 10.3390/polym14091899

**Published:** 2022-05-06

**Authors:** Diego Pugliese, Giulio Malucelli

**Affiliations:** 1 Politecnico di Torino, Department of Applied Science and Technology, and Local INSTM Unit, C.so Duca degli Abruzzi 24, 10129 Torino, Italy; diego.pugliese@polito.it; 2 Politecnico di Torino, Department of Applied Science and Technology, and Local INSTM Unit, Viale Teresa Michel 5, 15121 Alessandria, Italy

**Keywords:** UV-LED curable films, epoxy-acrylate resin, phosphate glass powder, thermal behavior, optical properties, flame retardance

## Abstract

In this work, we thoroughly investigate the effects of the incorporation of a phosphate glass micrometric powder on the morphology, as well as on the thermal, optical, mechanical and flame retardant properties of UV-LED curable acrylic films. To this aim, the filler loading was changed within 10 and 50 wt.%. UV-LED initiated curing was selected as a fast and reliable system, as the standard UV-curing process was not suitable because of the presence of the glass powder that decreased the quantum efficiency during the UV exposure, hence preventing the transformation of the liquid system into a solid network. The glass powder slightly increased the glass transition temperature of the acrylic network, hence showing a limited effect on the chain segments mobility; besides, increasing filler loadings were responsible for a progressive decrease of the transparency of films, irrespective of a marginal effect on their refractive index. Conversely, the presence of increasing amounts of phosphate glass improved the thermal and thermo-oxidative stability of the cured products. Besides, phosphate glass was capable of remarkably enhancing the flame retardance of the acrylic network at 50 wt.% loading, which achieved self-extinction in vertical flame spread tests (and was V-0 rated). This formulation, as assessed by forced-combustion tests, also displayed a remarkable decrease of peak of Heat Release Rate and Total Heat Release (by 44 and 33%, respectively) and of Total Smoke Release and Specific Extinction Area (by 53 and 56%, respectively). Further, the filler promoted an increase of the stiffness and surface hardness of the films, at the expense of a decrease in ductility. All these findings may justify the potential use of these composite films as flame retardant coatings for different flammable substrates.

## 1. Introduction

During the last 10 to 15 years, photoinduced polymerization (i.e., UV-curing) has become a reliable and efficient technique suitable for industrial scale applications, thanks to its high curing kinetics, low toxicity (because of the absence of VOC—Volatile Organic Compounds), energy saving (energy is necessary just to activate the reaction process) and limited environmental impact. All these features fully justify the use of UV-curing processes in such different fields, as printing inks, fast drying varnishes, protective coatings, printed circuit boards and optical fiber coatings, among a few to mention [1,2,3,4].

Despite of all these advantages, the standard UV-curing practice relies on the use of high-pressure Hg lamps as effective radiation sources. Conversely, the very high temperatures achieved during the irradiation process limit the appropriateness of the high-pressure Hg lamps for temperature-sensitive substrates, such as paper, wood, textiles and plastic films [5]. In addition, the release of high amounts of heat, together with the generation of ozone, significantly contributes to the increase of the environmental impact [6]. Quite recently, it has been demonstrated that it is possible to limit the heat release, while decreasing the energy consumption and keeping high curing efficacies by utilizing light emitting diodes (LEDs) as radiation sources, hence taking advantage of the so-called UV-LED curing processes [7,8,9]. In this way, it is possible to exploit a higher electrical-to-optical conversion efficiency with respect to the use of traditional UV-curing methods; further, UV-LED lamps do not emit in the infrared region, and therefore they are able to deliver a cool radiation without impacting on temperature-sensitive substrates [10].

At present, the UV-LED curing is primarily exploited for the preparation of photo-curable coatings or inks [11,12]: as a matter of fact, only a very limited number of research works specifically involving UV-LED curing has been addressed to the design, preparation and characterization of polymeric composite systems. In particular, one paper investigated the effect of the presence of 5 wt.% of phyllosilicate (Cloisite 30B) on the thermal and barrier properties of an UV-LED cured acrylic system [13]. It was found that the presence of the intercalated nanoclay accounted for an increase of the glass transition values of the polymer matrix that exhibited improved thermal and thermo-oxidative stability, as well as decreased permeability toward oxygen.

Besides, in a very recent paper, Wang and co-workers thoroughly investigated the effects of the “one pot” incorporation of different inorganic micro-fillers (namely, CaCO_3_, talc, and clay), up to about 50 wt.% loading, on the tribological and antibacterial features of UV-LED curable systems based on an epoxy-acrylate and trimethylolpropane triacrylate (used as reactive diluent) [14]. The UV-LED curing process allowed obtaining fully cured films having a nominal thickness of about 75 μm, with an exposure to a static UV-LED source of 6 s. The resulting films showed improved wear resistance, as well as antibacterial properties toward *E. coli* and *S. aureus* (bactericidal coefficient of approximately 99.5 and 97%, respectively), because of the presence of Ag nanoparticles in the UV-LED curable recipe. 

Composite films based on polymer matrices containing inorganic fillers (at micro- or nano-scale) have been intensively studied in the last decades, as the incorporation of an inorganic phase homogeneously dispersed within the polymer allows obtaining a final system with improved performances (i.e., enhanced thermal and/or thermo-oxidative stability, mechanical and tribological behavior, barrier properties toward different gases, electrical features…). In this context, the scientific literature is plenty of nice examples, including silica particles [15,16], polyhedral oligomeric silsesquioxanes (POSS) [17], nanoclays [18], bohemites [19], carbon nanotubes [20], and titania [21], among a few to mention.

Though in many cases the best overall behavior of the filled polymer systems (either thermoplastics or thermosets) is achieved when nano-sized fillers are incorporated [22], the use of micro-sized counterparts could be advantageous, especially when the dispersion of the nanofillers in the host polymer is difficult, because of severe reaggregation phenomena taking place during processing. Besides, micro-fillers may confer higher mechanical properties than nano-counterparts, as reported in the scientific literature [23,24]. 

In this context, micropowders of phosphate glasses may represent a good alternative to the aforementioned fillers, for the design of polymer composites with enhanced properties. Certainly, in recent years, phosphate glasses have demonstrated to be a sound alternative to silicate glasses as a host material for a wide range of photonic applications. In particular, their higher rare-earth ions solubility with respect to silicate glasses has allowed the development of more compact photonic devices, such as lasers and amplifiers [25,26]. Besides, the phosphate glasses are very well known for their homogeneity, good chemical durability and thermal stability and excellent optical properties [27]. If compared to silicate glasses, they show low glass transition (*T_g_*) (400–700 °C) and softening (*T_s_*) (500–800 °C) temperatures, which facilitate their processing and fabrication by melt-quenching technique [28]. Phosphate glasses have also been used as promising biomaterials because of their ability to dissolve completely in aqueous solutions into safe dissolutions, commonly found in the human body [29]. Further, another important feature of phosphate glasses refers to their thermal and mechanical strength, which allows the production of optical fibers that can be easily integrated in commercial systems based on silicate optical fiber components through fusion-splicing processes [30].

However, the potentialities of phosphate glasses are still not fully valorized: in particular, their incorporation in the form of micrometric powders into thermosetting polymer matrices has been investigated marginally and only for flame retardant purposes. 

In particular, Yu and co-workers [31] prepared a low-melting glass through hydrolytic polycondensation of phenyltriethoxysilane and a subsequent heat treatment; the so-obtained product was incorporated into a tetraphenylphosphonium-modified montmorillonite (5 wt.% loading)/epoxy system at different concentrations (namely, 5, 10 and 15 wt.%). The combination of the low-melting glass with the phyllosilicate turned out to enhance the flame retardance of the epoxy resin: as assessed in forced-combustion tests (carried out at 50 kW m^−2^), a significant decrease (by about 51% for the highest glass loading) of peak of Heat Release Rate was found and attributed to the formation of a molten continuous glass film exerting a thermal shielding effect on the surface of the irradiated samples.

In a further research effort, the same group [32] exploited the reaction between phenylphosphonic acid and methyltrichlorosilane or methyltriethoxysilane to obtain a low-melting glass containing P and Si elements. The glass was incorporated into tetraphenylphosphonium-modified montmorillonite (5 wt.% loading)/epoxy system at different concentrations (namely, 5, 10 and 15 wt.%). Again, the concurrent presence of the modified clay and the synthesized glass accounted for a decrease of Total Heat Release (up to 28%) and of peak of Heat Release Rate (by 48%) with respect to the unfilled cured epoxy resin, as assessed during forced-combustion tests (irradiative heat flux: 50 kW m^−2^).

Very recently, Liu and co-workers [33] thoroughly studied the flame retardant properties of low melting phosphate glasses/epoxy composites, in the presence of ammonium polyphosphate. The presence of the inorganic filler turned out to remarkably enhance the overall fire performances of the resulting composites, which achieved V-0 rating in vertical flame spread tests and, in forced-combustion tests, showed significantly decreased values of peak of Heat Release Rate (up to −45%) and Total Heat Release (up to −55%) with respect to the unfilled epoxy system.

To the best of our knowledge, the application of UV-LED curing processes to acrylic resins containing phosphate glass powders has never been investigated and reported in the scientific literature so far. Therefore, in the present work, we demonstrate the feasibility of the UV-LED curing process (performed in dynamic conditions, i.e., using a belt conveyor) for obtaining free standing epoxy-acrylate films containing different amounts (namely, 10, 20, 30, 40 and 50 wt.%) of phosphate glass powder and showing enhanced thermal and flame retardant features. To this aim, a commercially available epoxy-acrylate resin, namely bisphenol-A-ethoxylate-diacrylate, was selected as a model system. Then, the effect of the filler on the overall morphology, as well as on the thermal, optical, mechanical, and flame retardant behavior of the obtained composite films, was thoroughly studied and correlated with the phosphate glass powder loading, establishing some interesting structure-property relationships. 

## 2. Materials and Methods

### 2.1. Materials

**Figure 1:**
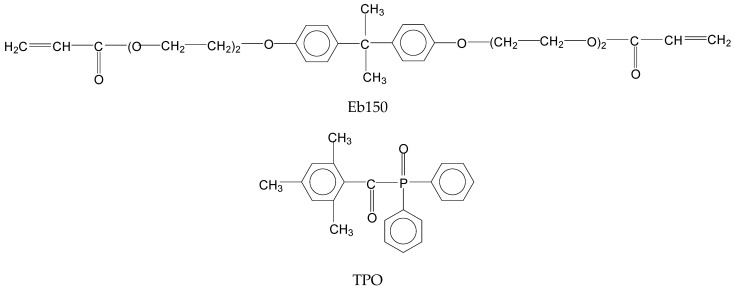
Chemical structures of Eb150 and TPO.

A commercially available epoxy-acrylate resin, Photomer 4028 (bisphenol-A-ethoxylate-diacrylate, bearing 4 ethylene oxide units, hereinafter coded as Eb150), and 2,4,6-Trimethylbenzoyl-diphenylphosphineoxide, hereinafter coded as TPO, were kindly supplied by IGM Resins (IGM, Mortara, Italy). TPO was employed as photoinitiator for the UV-LED curing process. The chemical structures of Eb150 and of TPO are presented in Figure 1.

The phosphate glass powder, with composition 65 P_2_O_5_—16 K_2_O—10 Al_2_O_3_—4 B_2_O_3_—5 MgO (in mol.%), was prepared by the traditional melt-quenching method. Briefly, a blend of oxides and carbonates was weighed and mixed within a dry box and the batched chemicals were melted in an alumina crucible at a temperature of 1320 °C under controlled atmosphere. After 1 h, the melt was quenched onto a cold aluminum plate and the resulting glass fragments were ground into fine micrometric powder (average size < 40 µm) by a 2 h ball-milling process (Pulverisette 0, Fritsch, Idar-Oberstein, Germany).

### 2.2. Preparation of the UV-LED Cured Films

The phosphate glass powder was dispersed into Eb150 at different concentrations (namely, 10, 20, 30, 40 and 50 wt.%) through mechanical stirring. Then, the photoinitiator (6 wt.%) was added to the UV-LED curable dispersions that were subsequently coated on glass plates using a wire-wound applicator (nominal thickness: 200 μm). Next, the coated glass plates underwent the UV-LED curing process, using a Heraeus Noblelight UV-LED NC1 unit (Heraeus Noblelight, Cambridge, UK), working in dynamic conditions (belt speed: 1 m min^−1^, which roughly corresponds to 2 s exposure), at 395 nm. The radiation intensity, measured with a Power Puck II^®^ radiometer (EIT, Sterling, VA, USA) on the sample surface, was around 4.8 W cm^−2^; the energy density (i.e., the dose) was about 10 J cm^−2^. After the UV-LED curing, free-standing films were peeled off from the glass substrate and utilized for the subsequent characterizations. Hereinafter, the prepared formulations will be coded as Eb150 (unfilled resin) and Eb150 + XX%G, where XX represents the weight percentage of phosphate glass (G) powder filled in the polymer matrix.

### 2.3. Characterization Techniques

A Perkin Elmer Spectrum 100 spectrometer (Shelton, CT, USA) equipped with an attenuated total reflection (ATR) diamond probe was employed for evaluating the completeness of the UV-LED curing reaction under the adopted experimental conditions for the UV-LED curing process. The FTIR spectra were recorded within 700 and 4000 cm^−1^, with 4 cm^−1^ resolution; 16 scans were collected for each spectrum.

The morphology of the prepared composite films was investigated by means of a Scanning Electron Microscope SEM Zeiss (Oberkochen, Germany; beam voltage: 20 kV) on the cross-sections of the investigated samples fractured in liquid nitrogen. Before the observations, the fracture surface of each sample was gold metallized to become electrically conductive.

Differential scanning calorimetry (DSC) analyses were performed using a QA1000 TA Instrument apparatus (TA Instrument Inc., Waters LLC, New Castle, DE, USA). All the experiments were performed under dry N_2_ gas (flow: 50 mL min^−1^); the samples (around 10 mg), placed in sealed aluminum pans, underwent the following cycle:

- heating from 0 to 160 °C at 10 °C min^−1^;
- cooling down to 0 °C at 10 °C min^−1^;
- final heating from 0 to 160 °C at 10 °C min^−1^.

The first heating scan was exploited for further confirming the completeness of the UV-LED curing reaction; glass transition temperature (*T_g_*) values were evaluated on the second heating scan, hence avoiding the superimposition of the *T_g_* with the enthalpy relaxation attributable to the non-equilibrium thermodynamic state, in which the macromolecules are frozen due to the fast UV-LED curing process, as already reported in the scientific literature [34].

A Discovery apparatus (TA Instrument Inc., Waters LLC, New Castle, DE, USA) was employed for assessing the thermal and thermo-oxidative stability of the cured films. Samples (about 10 mg) were placed in open alumina pans and heated up within 50 and 700 °C, with a heating rate of 10 °C min^−1^, under either nitrogen or air flow (35 and 25 mL min^−1^, respectively). *T_5%_* (i.e., the temperature, at which 5% weight loss takes place, identified as degradation onset) and *T_max_* values (i.e., the temperatures corresponding to the peaks appearing in dTG—derivative—curves) were calculated; besides, the final residue at 700 °C was measured. The experimental error was ±0.5 wt.% on mass and ±1 °C on temperature.

The reaction to an applied flame was assessed by means of UL 94 vertical burning tests according to IEC 60695-11-10 standard (sample dimensions: 100 × 50 × 0.2 mm^3^).

Cone calorimetry tests (Noselab Ats, Nova Milanese, Italy) were carried out to measure Time to ignition (TTI, s), Time to peak of Heat Release Rate (Time to pkHRR, s), peak of Heat Release Rate (pkHRR, kW m^−2^), Total Heat Release (THR, MJ m^−2^), Total Smoke Release (TSR, m^2^ m^−2^), Specific Extinction Area (SEA, m^2^ kg^−1^), CO/CO_2_ ratio and Residue mass (%) at the end of the test. The ISO 5660 standard was followed, applying an irradiative heat flux of 35 kW m^−2^. Specimens (100 × 100 × 0.2 mm^3^) were wrapped with an aluminum foil except for the irradiated sample surface and measured in horizontal position. At least three tests were performed for each composition, to provide reproducible and significant data and the results averaged.

For assessing the optical properties of the cured films, their transmittance spectra were measured at room temperature for wavelengths ranging from 200 and 900 nm using a double beam scanning spectrophotometer (UV-2600, Shimadzu, Columbia, MD, USA).

The refractive index (*n*) of the films was measured at 633 and 825 nm by prism-coupling technique (Metricon, model 2010, Pennington, NJ, USA). Ten scans were performed for each measurement and the estimated error of the measurement was ±0.001.

Tensile tests were carried out with an Instron 5966 dynamometer (Norwood, MA, USA). The measurements were performed at 1 mm min^−1^ crosshead speeds, using a 5 kN load cell and pneumatic grips. At least five specimens for each system were tested and the results averaged.

Pencil hardness tests were performed according to the ASTM D 3363-00 standard. 

## 3. Results and Discussion

### 3.1. FTIR-ATR Analyses

**Figure 2:**
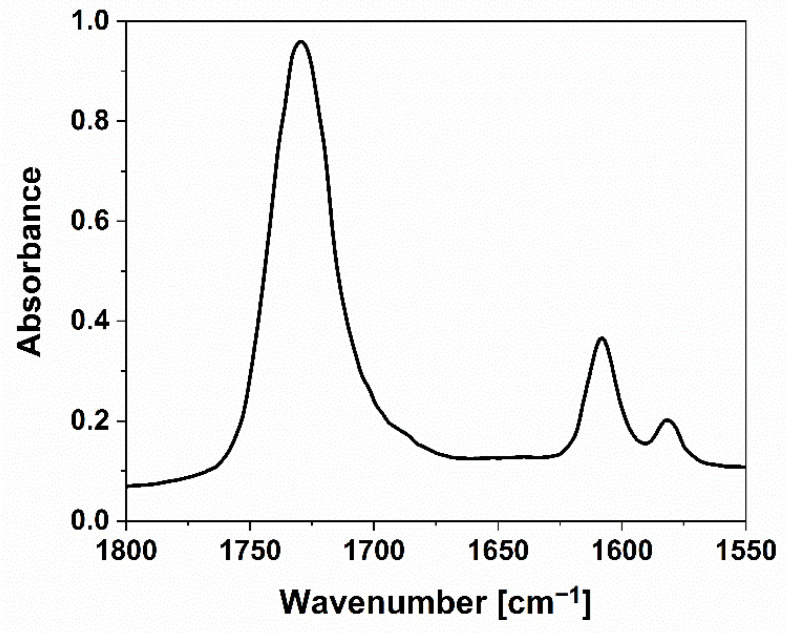
Typical FTIR-ATR spectrum of a UV-LED cured film containing 50 wt.% of phosphate glass powder (16 scans collected).

FTIR-ATR spectroscopy was exploited to verify the completeness of the curing process induced by the UV-LED radiation, according to the adopted experimental conditions (see Section 2.2). As an example, Figure 2 shows the typical FTIR-ATR spectrum of the UV-LED cured system containing the highest phosphate glass powder loading (i.e., 50 wt.%). The presence of an absorbance band at 1730 cm^−1^ is assigned to C=O; besides, the stretching vibration of the C=C double bonds is associated with the signal at 1635 cm^−1^ [35,36]. After the UV-LED curing process, the photoinitiator gives rise to the formation of active radicals that open the double bonds in the monomer, hence promoting the crosslinking reactions. As shown in Figure 2 for the composite film containing the highest phosphate glass loading, after the UV-LED curing process, the double bond peak totally disappears, hence confirming the completeness of the curing reaction, even in the presence of high filler amounts.

### 3.2. Morphology of the UV-LED Cured Films

**Figure 3:**
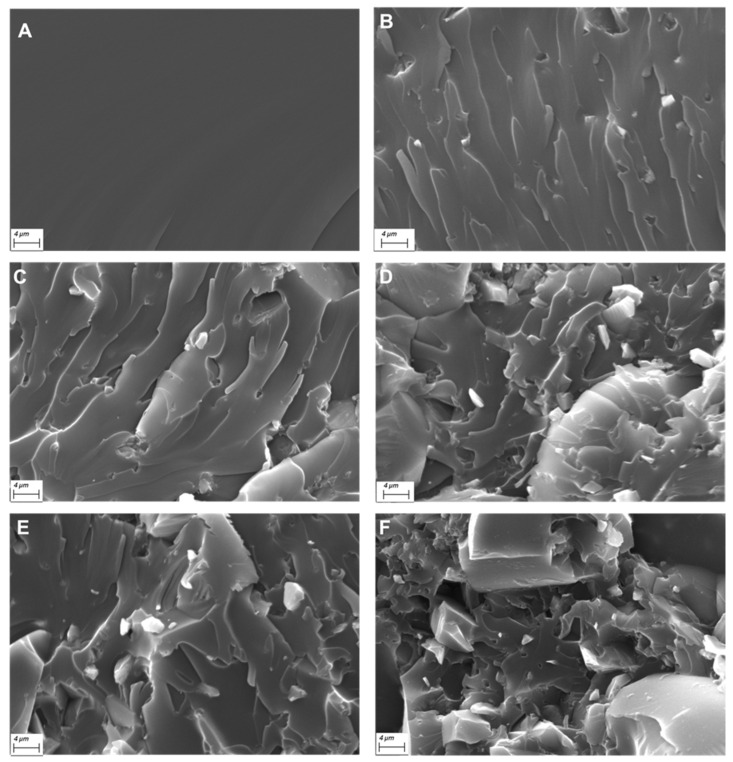
Typical SEM micrographs (at 5000×) of UV-LED cured films: Eb150 (**A**), Eb150 + 10%G (**B**), Eb150 + 20%G (**C**), Eb150 + 30%G (**D**), Eb150 + 40%G (**E**), Eb150 + 50%G (**F**).

Figure 3A–F shows the typical morphology of the prepared UV-LED cured films, as assessed by SEM analysis.

**Figure 4:**
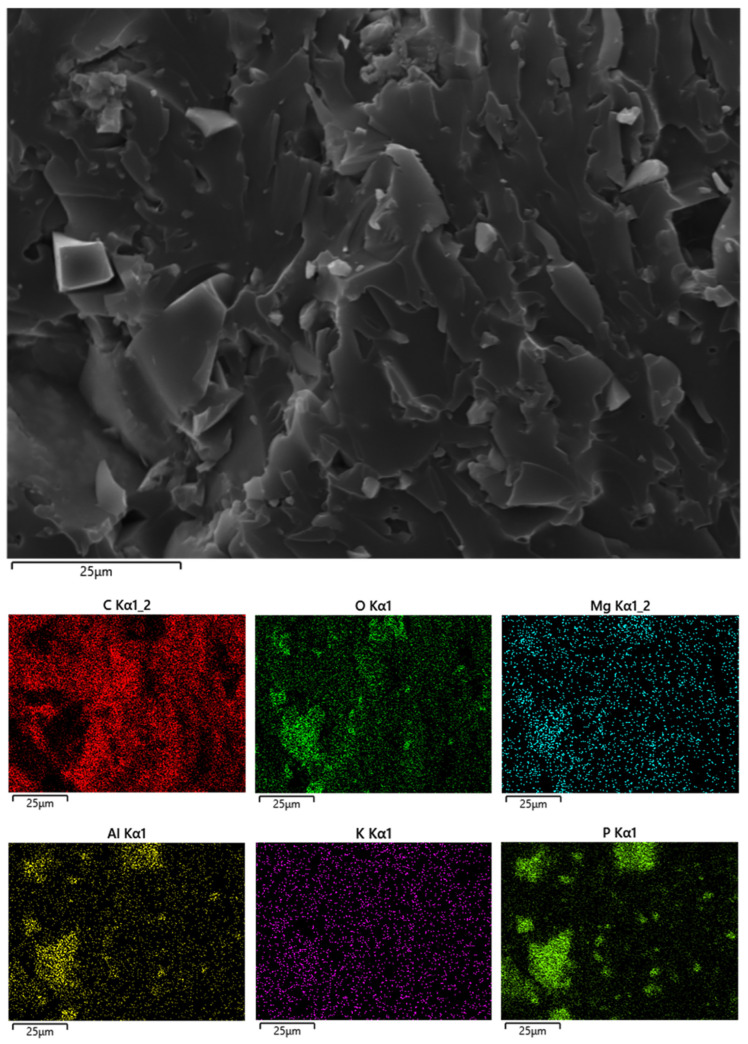
Elemental mapping for Eb150 + 40%G UV-LED cured film.

The unfilled UV-LED cured resin (Figure 3A) shows a very smooth fracture surface; the incorporation of increasing amounts of phosphate glass powder dramatically changes the morphology of the films, making their surface very rough. Besides, the distribution of the filler is quite homogeneous within the hosting polymer matrix: irregular particles (size: about few microns, though some larger aggregates are present) are well dispersed within the polymer phase, as also further supported by Energy Dispersive X-ray (EDX) analyses (as an example, the typical maps of the main elements of the phosphate glass are shown in Figure 4 for the system containing 40 wt.% of filler).

### 3.3. Thermal Behavior

DSC analyses were performed in order (i) to further support the completeness of the UV-LED curing process and (ii) to evaluate the glass transition temperature (*T_g_*) values and their possible changes in the presence of increasing phosphate glass powder loadings. For this purpose, a heating/cooling/heating thermal cycle was conceived as detailed in the Materials and Methods Section. The first heating up was chosen only for confirming the absence of exothermal peaks that would have been a clear indication of incompleteness of the curing process, hence further supporting the FTIR-ATR outcomes.

Table 1 lists the *T_g_* values of the UV-LED cured films, as measured on the second heating up. It is worthy to note that the presence of increasing filler loadings slightly affects the glass transition temperature of the polymer network, which rises from 58.5 °C (unfilled cured system) to 65.1 °C (for the system containing 50 wt.% of phosphate glass powder). The observed *T_g_* increase indicates a moderate effect exerted by the filler particles on the mobility of the polymer segments in the formed 3-dimensional (3D) network, as already reported in the literature [37].

Then, the thermal and thermo-oxidative stability of the UV-LED cured systems was evaluated by means of thermogravimetric analyses carried out in nitrogen and air atmospheres, respectively. The obtained data are collected in Table 2.

In nitrogen, Eb150 shows a single degradation step, within 350 and 475 °C, during which a progressive fragmentation of the polymer network occurs. The incorporation of increasing amounts of phosphate glass powder remarkably increases the degradation onset (calculated as *T_5%_*, i.e., the temperature at which 5% of sample mass is lost), while does not affect the *T_max_* values (that remain at about 440 °C, irrespectively of the filler loading). This behavior is quite common for the micro-sized inorganic fillers, as already reported in the literature [38,39,40].

In air, the thermo-oxidative degradation of the UV-LED cured acrylic resin takes place according to two consecutive steps: the former, occurring between 350 and 475 °C, corresponds to the main degradation of the polymer network; the latter (in between 525 and 650 °C) refers to the oxidation of the products obtained from the previous step. Though the degradation onset in air is anticipated with respect to that in nitrogen because of the oxidative atmosphere, the presence of increasing amounts of the filler shifts the *T_5%_* values toward higher temperatures, hence indicating a protective effect exerted by the ceramic filler that slows down the degradation phenomena. Once again, as already observed in nitrogen, *T_max_* values are not influenced by the presence of the phosphate glass powder and its loading. Besides, the observed residues at the end of the thermogravimetric analyses indicate a slight char-forming effect of the ceramic filler.

### 3.4. Fire Behavior

It is well reported in the scientific literature that the incorporation of any non-combustible filler will decrease the flammability of polymer systems, as the filler decreases the total amount of fuel, as well as the diffusion rate of oxygen into, and of the fuel from the polymer bulk, while rising the heat capacity, and reflectivity [41]. In particular, phosphate glasses, upon heating, may favor the creation of an inert layer on the surface of the decomposing polymer substrate: this layer acts as a thermal protective shield that slows down the radiant heat and mass transfer phenomena occurring during the application of an irradiative heat flux or a flame. The potential flame retardant effectiveness of phosphate glass has been assessed through flammability (i.e., vertical flame spread tests) and forced-combustion tests, using a cone calorimetry apparatus. 

As far as flammability tests are concerned, all the UV-LED cured films were not classifiable, except for Eb150 + 50%G (i.e., the film containing the highest phosphate glass powder loading), which achieved self-extinction and was V-0 rated. These findings clearly indicate that the use of phosphate glasses alone (i.e., not in combination with other flame retardant additives, as reported in the literature [33]) allows achieving good flame retardant performances at high filler loadings only; on the other hand, 50 wt.% of filler well performs, but may worsen the mechanical behavior (as described in Section 3.6), too much increasing the stiffness of the polymer matrix, while dramatically decreasing its ductility and impact resistance [42,43].

To get further information about the flame retardant behavior of the designed films, cone calorimetry tests were carried out, exposing the samples to 35 kW m^−2^ irradiative heat flux. Table 3 lists the most important thermal and smoke parameters obtained from these analyses.

**Figure 5:**
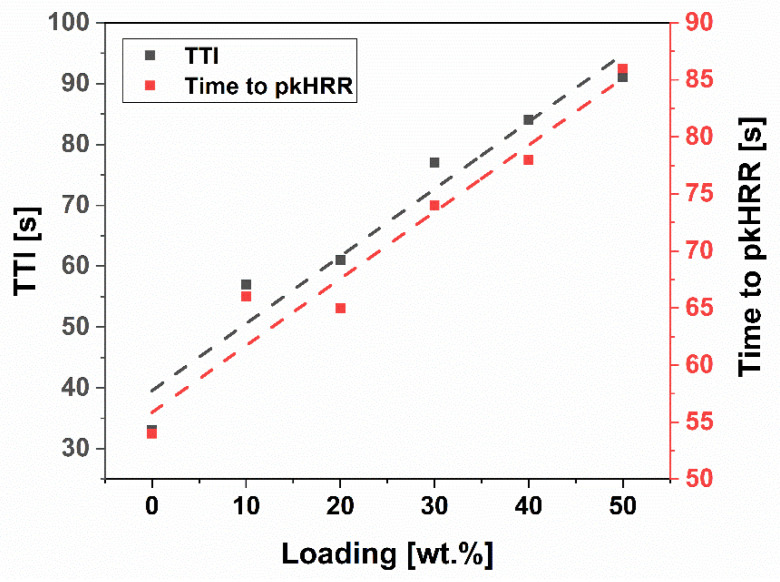
Time to Ignition and Time to peak of Heat Release Rate as a function of the glass phosphate powder loading. The square symbols are experimental values, while the dashed lines are linear fitting curves.

First, it is noteworthy that the inorganic filler increases both Time to Ignition (TTI) and Time to peak of Heat Release Rate (time to pkHRR) of the cured polymer matrix; this finding is very common in polymer systems containing inorganic fillers [41]. In the present work, it is expected that the thermal inertia of phosphate glass powder will delay the achievement of the critical temperature value for igniting the specimen. Besides, the higher is the filler loading, the longer is the time needed for igniting the sample and for achieving the peak of Heat Release Rate: the trend of the two cone parameters is quite linear with the filler loading, as depicted in Figure 5.

**Figure 6:**
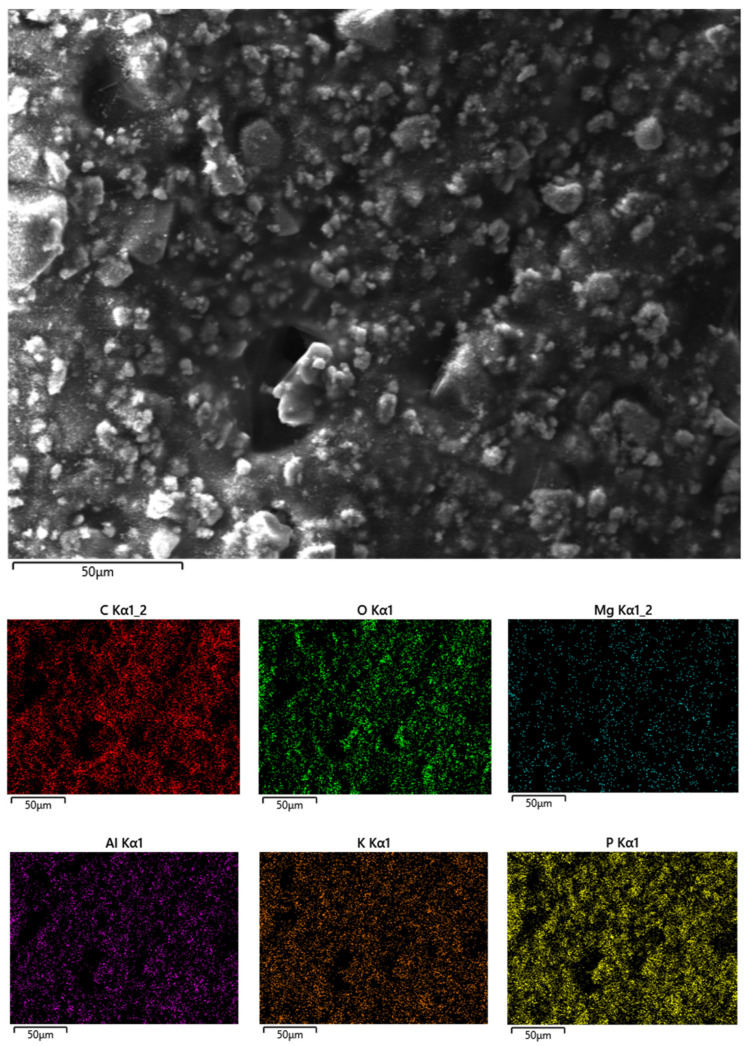
Elemental mapping for the Eb150 + 30%G residue after cone calorimetry tests.

Besides, the incorporation of increasing amounts of filler causes a remarkable decrease of both peak of Heat Release Rate and Total Heat Release, which are lowered up to about 44 and 33%, respectively, when 50 wt.% of phosphate glass powder is included in the thermosetting matrix. This finding further confirms the thermal shielding effect exerted by the filler, which also shows very good performances as smoke suppressant, as witnessed by the significant lowering of Total Smoke Release (TSR) and Specific Extinction Area (SEA). In addition, the slight increase of CO/CO_2_ ratio suggests that the selected filler predominantly acts in the condensed phase, as also advised by the high residues found at the end of the test. The morphology of these latter, as assessed by SEM-EDX analyses, reveals the presence of a “hybrid” co-continuous char/glass structure: as an example, Figure 6 displays the morphology of the burnt surface and the typical maps of the main elements present in the residue for the system containing 30 wt.% of filler.

### 3.5. Optical Properties

**Figure 7:**
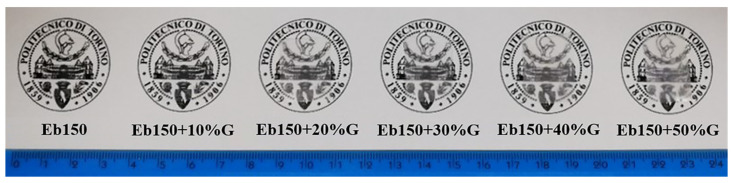
Pictures of UV-LED cured films prepared with different loadings of phosphate glass particles.

**Figure 8:**
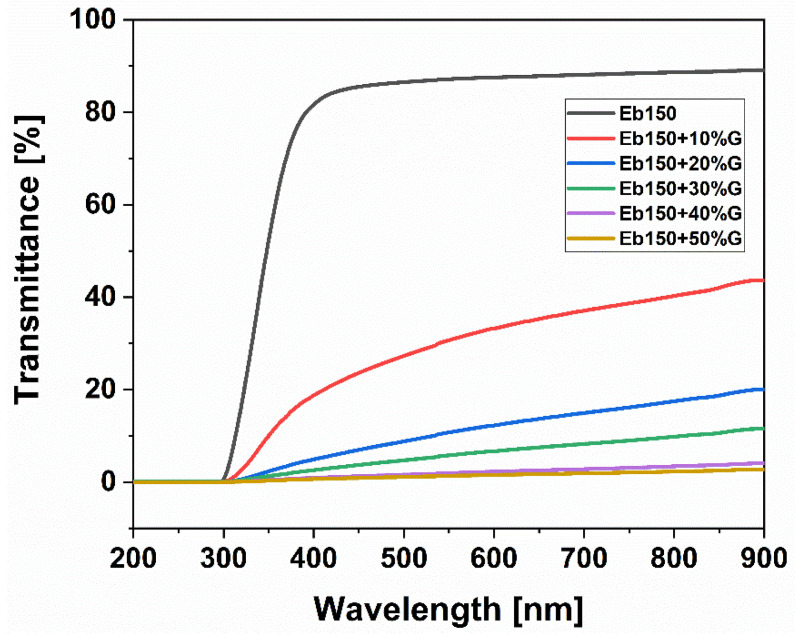
Transmittance spectra of UV-LED cured films loaded with different amounts of phosphate glass particles.

Figure 7 shows the photos of the UV-LED cured films at different phosphate glass loadings ranging from 0 to 50 wt.%. As expected, the transparency of the samples was found to decrease progressively as the amount of glass dispersed in the epoxy-acrylate resin increased, due to scattering phenomena exerted by the dispersed inorganic phase. This result is in agreement with the transmittance (*T*%) spectra shown in Figure 8 in the wavelength region 200–900 nm. Pure Eb150 film showed *T* = 87.7% at the red-light wavelength of 633 nm, however, this value decreased to 34.7, 13.2, 7.2, 2.4 and 1.7% after the incorporation of 10, 20, 30, 40 and 50 wt.% of phosphate glass, respectively.

Table 4 collects the refractive index values at 633 and 825 nm of the UV-LED cured films prepared with different phosphate glass particle loadings. Contrary to the trend shown by the transmittance, the addition of increasing amounts of phosphate glass particles did not lead to significant changes in the refractive index of the UV-LED cured films. This difficulty in matching the refractive index of a polymer matrix with that of an inorganic filler has already been reported in the recent literature [44].

### 3.6. Mechanical Behavior

To assess the effect of the different phosphate glass powder loadings on the mechanical behavior of the UV-LED cured films, both tensile and pencil hardness tests were performed. The results are collected in Table 5.

As expected, the incorporation of the filler increased the stiffness of the UV-LED cured films, while remarkably decreasing their ductility, hence making the UV-LED cured films very brittle. This behavior is very common for polymer systems filled with high loadings of inorganic fillers [37]. Finally, the presence of increasing phosphate glass powder loadings progressively increased the surface hardness of the films, as shown by the continuous rise of pencil hardness values, from 2B (unfilled Eb150) to 3H (UV-LED system containing the highest filler amount). 

## 4. Conclusions

In the present work, the effects of the incorporation of different amounts of phosphate glass micrometric powder (ranging from 10 to 50 wt.%) on the morphology, thermal, optical, mechanical and flame retardant properties of a UV-LED curable acrylic resin have been thoroughly investigated. 

The UV-LED curing process allowed obtaining fully cured films, regardless of the filler loading, as assessed by FTIR-ATR and DSC analyses. 

The filler was found homogeneously dispersed in the polymer matrix, even at very high loadings; only few aggregates, not exceeding 40-µm size, were observed by SEM analyses. 

The presence of the phosphate glass powder slightly affected the glass transition temperature values of the polymer network, hence indicating a limited effect on the mobility of the macromolecular chain segments: indeed, the *T_g_* shifted from 58.5 (unfilled system) up to 65.1 °C (films containing 50 wt.% of filler). Conversely, the thermal and thermo-oxidative stability of the obtained films was improved by the incorporation of increasing filler loadings, hence highlighting a protective effect exerted by the ceramic filler that slowed down the degradation phenomena. 

The selected filler was found to significantly enhance the flame retardant features of the UV-LED cured films. In particular, at the highest filler loading, the films were self-extinguishing, and V-0 rated in vertical flame spread tests. In addition, as revealed by forced combustion tests performed under 35 kW m^−2^ irradiative heat flux, the phosphate glass powder was responsible for a remarkable increase of Time to Ignition and Time to peak of Heat Release Rate (both linearly increasing with increasing the filler loading), as well as for a significant continuous decrease of Heat Release Rate and Total Heat Release at increasing filler loadings. Besides, the selected filler acted as an efficient smoke suppressant, lowering both Total Smoke Release and Specific Extinction Area (up to 53 and 56%, respectively, for the system containing the highest filler loading). These findings were attributed to the thermal shielding effect in the condensed phase exerted by the phosphate glass powder, which, upon the exposure to an irradiative heat flux or a flame, formed a sort of “hybrid” co-continuous char/glass structure on the surface of the degrading film.

As far as the optical properties are considered, the glass phosphate powder was responsible for an important decrease of the transparency of UV-LED cured films over the visible wavelength range, regardless of a very limited influence on their refractive index. 

Finally, the presence of increasing filler loadings was found to rise the stiffness and the surface hardness of the prepared films, notwithstanding a significant lowering of their ductility, as assessed by tensile and pencil hardness tests.

## Figures and Tables

**Table 1:** Glass transition temperatures of UV-LED cured films.

Sample	*T_g_* [°C]
Eb150	58.5
Eb150 + 10%G	59.3
Eb150 + 20%G	59.5
Eb150 + 30%G	61.0
Eb150 + 40%G	62.1
Eb150 + 50%G	65.1

**Table 2:** Thermal and thermo-oxidative stability of UV-LED cured films.

*Atmosphere: Nitrogen*						
Sample	*T_5%_* [°C]	*T_max 1_* * [°C]	Residue @ *T_max 1_* [%]	*T_max 2_* * [°C]	Residue @ *T_max 2_* [%]	Residue @ 700 °C [%]
Eb150	299	441	40.0	-	-	4.1
Eb150 + 10%G	312	439	48.8	-	-	14.3
Eb150 + 20%G	319	440	53.6	-	-	24.4
Eb150 + 30%G	331	439	60.1	-	-	34.9
Eb150 + 40%G	369	435	67.3	-	-	44.2
Eb150 + 50%G	377	433	73.1	-	-	53.8
***Atmosphere: Air***						
**Sample**	***T_5%_*** **[°C]**	***T_max 1_*** * **[°C]**	**Residue** **@ *T_max 1_* [%]**	***T_max 2_*** * **[°C]**	**Residue** **@ *T_max 2_* [%]**	**Residue** **@ 700 °C [%]**
Eb150	295	430	48.3	559	8.1	0.0
Eb150 + 10%G	301	431	50.4	553	27.2	14.0
Eb150 + 20%G	308	430	52.3	549	34.4	23.8
Eb150 + 30%G	326	433	54.1	556	40.1	34.2
Eb150 + 40%G	330	431	64.2	547	47.8	43.3
Eb150 + 50%G	345	431	74.6	547	56.4	53.7

* From dTG (i.e., derivative curves).

**Table 3:** Results from cone calorimetry tests performed on UV-LED cured films.

Sample	TTI [s]	Time to pkHRR [s]	pkHRR [kW m^−2^]	THR [MJ m^−2^]	TSR [m^2^ m^−2^]	SEA [m^2^ kg^−1^]	CO/CO_2_	Residue Mass [%]
Eb150	33	54	297	4.32	260	1007	0.40	1.7
Eb150 + 10%G	57	66	277	4.09	227	980	0.48	16.2
Eb150 + 20%G	61	65	212	4.24	215	875	0.46	27.4
Eb150 + 30%G	77	74	200	3.17	176	628	0.46	36.8
Eb150 + 40%G	84	78	182	2.98	145	534	0.47	47.7
Eb150 + 50%G	91	86	166	2.90	123	442	0.47	59.3

**Table 4:** Refractive index of UV-LED cured films at 633 and 825 nm.

Sample	*n* @ 633 nm ± 0.001	*n* @ 825 nm ± 0.001
Eb150	1.565	1.556
Eb150 + 10%G	1.568	1.559
Eb150 + 20%G	1.566	1.554
Eb150 + 30%G	1.563	1.556
Eb150 + 40%G	1.564	1.554
Eb150 + 50%G	1.563	1.554

**Table 5:** Young’s modulus, elongation at break and pencil hardness values for the investigated UV-LED cured systems.

Sample	Young’s Modulus [MPa]	Elongation @break [%]	Pencil Hardness
Eb150	2280 ± 65	13.0 ± 0.6	2B
Eb150 + 10%G	2455 ± 88	8.4 ± 0.5	B
Eb150 + 20%G	2870 ± 84	7.1 ± 0.7	HB
Eb150 + 30%G	3005 ± 96	5.3 ± 1.0	H
Eb150 + 40%G	3280 ± 112	3.4 ± 0.9	2H
Eb150 + 50%G	3450 ± 127	2.8 ± 0.9	3H

## Data Availability

Not applicable.
